# Synergy of Peptide and Sugar in *O*-GlcNAcase Substrate Recognition

**DOI:** 10.1016/j.chembiol.2012.01.011

**Published:** 2012-02-24

**Authors:** Marianne Schimpl, Vladimir S. Borodkin, Lindsey J. Gray, Daan M.F. van Aalten

**Affiliations:** 1 Division of Cell Signalling & Immunology, College of Life Sciences, University of Dundee, Dundee DD1 5EH, Scotland

## Abstract

Protein *O*-GlcNAcylation is an essential reversible posttranslational modification in higher eukaryotes. *O*-GlcNAc addition and removal is catalyzed by *O*-GlcNAc transferase and *O*-GlcNAcase, respectively. We report the molecular details of the interaction of a bacterial *O*-GlcNAcase homolog with three different synthetic glycopeptides derived from characterized *O*-GlcNAc sites in the human proteome. Strikingly, the peptides bind a conserved *O*-GlcNAcase substrate binding groove with similar orientation and conformation. In addition to extensive contacts with the sugar, *O*-GlcNAcase recognizes the peptide backbone through hydrophobic interactions and intramolecular hydrogen bonds, while avoiding interactions with the glycopeptide side chains. These findings elucidate the molecular basis of *O*-GlcNAcase substrate specificity, explaining how a single enzyme achieves cycling of the complete *O*-GlcNAc proteome. In addition, this work will aid development of *O*-GlcNAcase inhibitors that target the peptide binding site.

## Highlights

► Multiple *O*-GlcNAc peptides bind OGA with similar orientation and conformations ► OGA interacts with the peptide backbone of substrates, not with side chains ► Intramolecular hydrogen bonds affect substrate conformation and *K*_m_ of glycopeptides ► Different OGA inhibitors display varying levels of peptide mimicry

## Introduction

Posttranslational modification of serines/threonines on intracellular eukaryotic proteins with *O-*linked *N*-acetylglucosamine (*O*-GlcNAc) is involved in numerous cellular processes such as transcription, cell cycle progression, and signal transduction (Hart et al., 2007; Love and Hanover, 2005). More than 1,000 proteins are known to be *O*-GlcNAcylated, and crosstalk with protein phosphorylation is believed to be extensive (Wang et al., 2010). Reversible protein *O*-GlcNAcylation is achieved by the action of two enzymes, *O*-GlcNAc transferase (OGT) and *O*-GlcNAcase (OGA). No precise *O*-GlcNAcylation sequence motif (sequon) has so far been defined, although promising site mapping data/tools have recently been reported (Chalkley et al., 2009; Wang et al., 2010; Zachara and Hart, 2004). OGT possesses an unusual N terminus, consisting of up to 13.5 tetratricopeptide repeats (TPRs) that are thought to play a role in recognition of intact protein substrates (Clarke et al., 2008; Iyer and Hart, 2003; Lubas and Hanover, 2000). Although recent studies have reported the structure of a bacterial OGT homolog (Clarke et al., 2008; Martinez-Fleites et al., 2008) and the structure of hOGT in complex with a peptide (Lazarus et al., 2011), the molecular mechanisms through which these TPRs contribute to selectivity of *O*-GlcNAc transfer are not yet understood.

The enzyme that removes *O*-GlcNAc, OGA, is a 103 kDa enzyme with two domains: an N-terminal hydrolase catalytic domain belonging to CAZy family GH84 (Henrissat and Davies, 1997) and a C-terminal domain that has been proposed to possess histone acetyltransferase activity (Gao et al., 2001; Toleman et al., 2004). Although human OGA (hOGA) can be expressed and purified yielding samples suitable for biochemical studies, attempts to crystallize the protein have so far failed. We recently used an apparent OGA homolog from *C. perfringens* (*Cp*OGA) to provide insights into the OGA structure (Rao et al., 2006), in parallel with a study on a similar enzyme from *B. thetaiotaomicron* (Dennis et al., 2006). These structures have identified the active site, which is almost fully conserved with hOGA, and have revealed the molecular details of the interaction with the GlcNAc sugar (Dennis et al., 2006; Rao et al., 2006). Complexes of these enzymes with widely used inhibitors of hOGA have facilitated structure-based design of the potent and selective thiazoline/GlcNAcstatin-based hOGA inhibitors (Dorfmueller et al., 2010; Yuzwa et al., 2008). Strikingly, both these bacterial enzymes were shown to possess *O*-GlcNAcase activity toward a broad spectrum of *O*-GlcNAc proteins in human cell lysates (Dennis et al., 2006; Yuzwa et al., 2008). Similar to OGT, it is not clear whether hOGA possesses a (glyco)peptide sequence preference, or how the enzyme binds glycopeptides and catalyzes their *O*-GlcNAc removal. Thus, the currently available structural information for OGT and OGA does not explain the molecular mechanisms of their interactions with protein substrates, and this limits our understanding of regulation of *O*-GlcNAc turnover and cycling rates. We investigated how OGA interacts with glycopeptide substrates, revealing that intramolecular interactions in the substrate may affect binding to the OGA active site, and elucidating how OGA achieves *O*-GlcNAc removal from *O*-GlcNAc sites in a sequence-independent manner.

## Results and Discussion

### *O*-GlcNAcase Glycopeptide Hydrolysis Is Independent of Peptide Length

**Figure 1:**
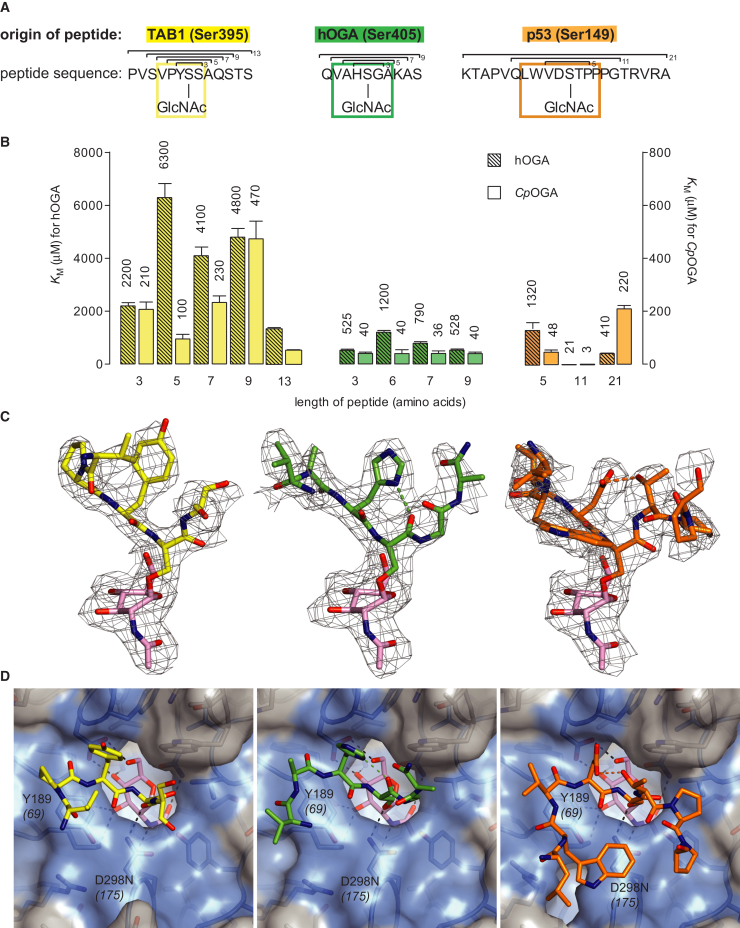
OGA Binds Different *O*-GlcNAc Glycopeptides with Similar Conformation but Different Affinities (A) Origin and sequence of *O*-GlcNAc peptides. The sequence of the longest peptides used is given, with residues observed in the crystal structure highlighted by colored boxes, and the shorter peptides indicated by brackets. (B) *K*_m_ of glycopeptide substrates for hOGA (displayed by filled bars [values for the three shortest hOGA- and TAB1-derived peptides reproduced from Schimpl et al., 2010]) and *Cp*OGA (unfilled bars, right y axis) as determined by substrate competition assay with error bars representing the error of curve-fit (see Experimental Procedures for details). (C) Conformation of *O*-GlcNAc peptides bound in the active site of *Cp*OGA as determined by X-ray crystallography. Peptides are shown as sticks with colored carbons (TAB1 peptide, yellow; hOGA peptide, green; p53 peptide, orange), with the GlcNAc sugar highlighted by pink carbons. Unbiased |F_o_|−|F_c_|,Φ_calc_ electron density (i.e., before addition of any glycopeptide model) is shown in gray (contoured at 2.25 σ). Intramolecular hydrogen bonds are shown by dashed lines. (D) *O*-GlcNAc peptides (sticks) in complex with *Cp*OGA D298N (surface representation). Sequence conservation between hOGA and *Cp*OGA is indicated by blue shading of identical residues on the molecular surface. The catalytic acid Asp298 (mutated to Asn) and the conserved Tyr198 are labeled, with corresponding residue numbers for hOGA given in brackets. See also Figures S1 and S2.

Although the structure of human OGA (hOGA) is not available, high-resolution structures of a homologous protein, NagJ, from *C. perfringens* have been reported, including complexes with the hOGA inhibitors PUGNAc (Rao et al., 2006) and GlcNAcstatin (Dorfmueller et al., 2006). Although it is unclear whether *Cp*OGA is a physiological OGA, it shows significant in vitro *O*-GlcNAcase activity on *O*-GlcNAc proteins in lysates from human cell lines and possesses a putative substrate-binding groove conserved with hOGA (Rao et al., 2006; Schimpl et al., 2010). Here we report that *Cp*OGA, like hOGA (Schimpl et al., 2010), possesses activity toward synthetic glycopeptides derived from validated *O*-GlcNAc sites in the human proteome, namely p53 (Ser149 [Yang et al., 2006]), TAK1-binding protein 1 (TAB1, Ser395 [Schimpl et al., 2010]), and hOGA itself (Ser405 [Lazarus et al., 2006]) (Figure 1). The Michaelis constants (*K*_m_) of these glycopeptides are consistently lower for *Cp*OGA and correlate with the values observed for hOGA (*r* = 0.91; see Figure S1 available online), suggesting that the bacterial enzyme is a suitable model for understanding hOGA-substrate interactions. Interestingly, there is no correlation between *K*_m_ for either enzyme and glycopeptide length, suggesting that the range in *K*_m_s observed for the different peptides (3-470 μM for *Cp*OGA, 21-6,300 μM for hOGA; Figure 1B) must stem from structural/sequence properties near the *O*-GlcNAc site.

### OGA Binds Different *O*-GlcNAc Peptides with Similar Conformations

To investigate the molecular basis of this substrate specificity, we exploited the catalytic acid mutant of *Cp*OGA, D298N, which is inactive yet unaffected in its ability to bind substrate (Rao et al., 2006). We generated an alternative *Cp*OGA crystal form with a highly accessible active site (see Figure S2) and determined the structures of *Cp*OGA D298N in complex with the p53-, TAB1-, and hOGA-derived *O*-GlcNAc peptides. Synchrotron diffraction experiments resulted in clear unbiased electron density difference maps that defined the conformations of all three glycopeptides (Figure 1C). These complexes define the molecular basis of how OGA recognizes both the sugar and protein components of physiologically relevant substrates. The *O*-GlcNAc sugar occupies the same position in all three structures (maximum atomic shift of 0.2 Å). It is tethered by extensive hydrogen bonding with residues that are identical between *Cp*OGA and hOGA (Figure 1D) and adopts the ^1,4^*B* boat conformation predicted from mechanistic studies and observed in complexes with pseudosubstrates (Macauley and Vocadlo, 2010; Macauley et al., 2005; Rao et al., 2006). Compatible with the proposed substrate-assisted catalytic mechanism, the carbonyl oxygen approaches the anomeric carbon to within 3.0 Å, poised for nucleophilic attack and in-line displacement of the glycosidic oxygen (angle of 164°).

### Glycopeptide Substrate Recognition Involves Backbone Contacts

Strikingly, the backbones of all three glycopeptides run in the same direction (Figure 1D), together defining the −4 through +3 subsites, and adopt similar conformations near the *O*-GlcNAc site (maximum Cα shift of 1.8 Å for the −2 to +1 subsites). Notably, all side chains point away from the surface of the enzyme (apart from Trp146 in the p53 peptide, which appears to stack with Asn298 of *Cp*OGA D298N), explaining how a single OGA enzyme is able to recognize >1,000 *O*-GlcNAc proteins. Hydrophobic stacking of the solvent-exposed Tyr189 (Tyr69 in hOGA) aromatic side chain with the −1 and −2 peptide bonds contributes a major amount (∼30% of the buried surface) of the interaction between the enzyme and the peptide component of the glycopeptides (Figure 1D). To test the contribution of this interaction to substrate binding, we mutated the corresponding Tyr69 in the human enzyme to Ser, Lys, Gln, and Phe and determined the *K*_m_ values for several substrate *O*-GlcNAc peptides (Table 1). Only the Y69F mutant shows no loss in activity, indicating that the hydrophobic stacking interaction is essential for OGA activity.

### Intramolecular Hydrogen Bonds Affect Substrate Conformation and K_m_

All three glycopeptides adopt a “V-shaped” conformation that allows the sugar to penetrate the OGA active site. Interestingly, for two of the peptides, this conformation appears to be stabilized by intramolecular hydrogen bonds (Figure 1C). The hOGA-derived glycopeptide forms a hydrogen bond between the histidine in the −1 subsite and the backbone carbonyl oxygen of the *O*-GlcNAc serine (Figure 1C). For the p53-derived glycopeptide, a hydrogen bond is observed between the aspartic acid in the −1 subsite and the threonine in the +1 subsite (Figure 1C). Such intramolecular interactions may stabilize the OGA-bound conformation of these glycopeptides, explaining the significantly lower *K*_m_s compared to the TAB1-derived peptide. We tested this hypothesis by designing glycopeptide sequences that either disrupted (in case of the p53-derived peptide) or introduced (in case of the TAB1-derived peptide) such intramolecular interactions (see Table 2). Indeed, introducing a hydrogen bond acceptor in the TAB1 peptide leads to a 5-fold decrease in *K*_m_ for hOGA, whereas disrupting the hydrogen bond in the p53-derived peptide, either by removing the hydrogen bond donor or acceptor, leads to an 85-fold increase in *K*_m_. Thus, although OGA substrate recognition does not appear to involve direct recognition of specific residues proximal to the *O*-GlcNAc site, subtle conformational effects appear to tune substrate recognition. It is possible that sequence-dependent stabilization of a specific backbone conformation around the *O*-GlcNAc site could give rise to differential persistence/cycling rates for individual *O*-GlcNAc sites in the human proteome.

### O-GlcNAcylation of p53 Involves Limited Conformational Change around the Acceptor Serine

**Figure 2:**
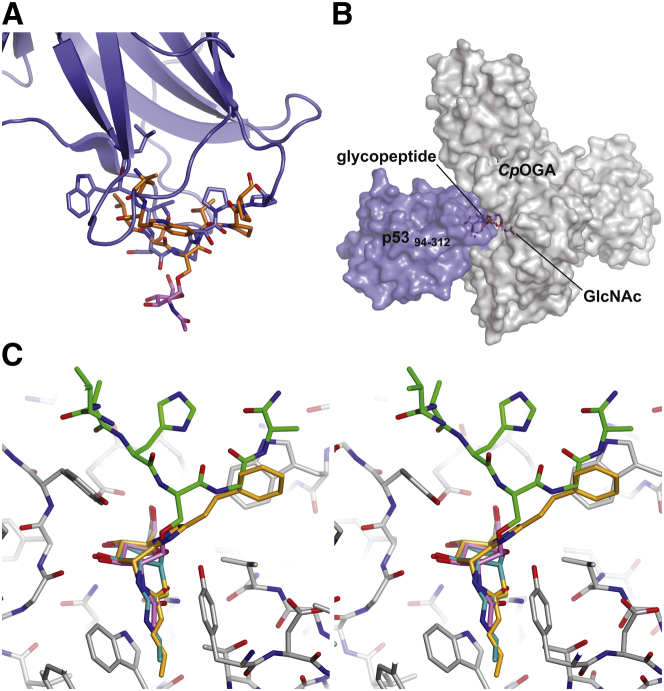
Comparison of Glycopeptide Binding with the Native p53 Conformation and Inhibitor Complexes (A) Superposition of the p53-derived glycopeptide structure as observed in the complex with *Cp*OGA D298N (orange and pink sticks) with the corresponding region of the p53 DNA binding domain crystal structure (Cho et al., 1994) (blue sticks and cartoon; Protein Data Bank ID 1tup). (B) Macromolecular model of *Cp*OGA (gray) and the p53 DNA binding domain (blue) in surface representation. (C) Comparison of substrate and inhibitor binding modes (divergent stereo image). *Cp*OGA (gray sticks) is shown in complex with the hOGA-derived substrate glycopeptide (green and pink sticks), *Cp*OGA-GlcNAcstatin G (Dorfmueller et al., 2010) (orange sticks), and with the thiazoline-derivative thiamet-G (blue sticks, obtained by superposition with the GH84 enzyme from *B. thetaiotaomicron*; Protein Data Bank ID 2vvn) (Yuzwa et al., 2008).

There are currently no available structures of *O*-GlcNAc glycoproteins, and *O*-GlcNAc-sites on structurally characterized proteins appear to reside in disordered/structurally undefined regions, limiting our understanding of the conformational changes induced by protein *O*-GlcNAcylation, or how these proteins would interact with OGA/OGT. A notable exception is the tumor suppressor protein p53, where the reported Ser149 *O*-GlcNAc site resides in a loop that is fully defined in the crystal structure of the p53 DNA binding domain (Cho et al., 1994). Ser149 lies at the tip of this loop, projecting out into the solvent. Comparing the structure of this loop in the p53 DNA binding domain structure with the glycosylated form reported here, it is apparent that the overall trajectory of the loop is approximately conserved between the two conformations of the peptide (Figure 2A; average Cα shift of 2.2 Å), although a number of side chain flips are observed (in particular Trp146). This superposition can also be expanded to the *Cp*OGA and p53 proteins, yielding a model of an *O*-GlcNAcase-glycoprotein substrate complex (Figure 2B), with p53 and its Ser149 loop occupying the OGA putative substrate binding site. Further work will be needed to establish how regions beyond the immediate vicinity of the *O*-GlcNAc site will contribute to the interaction of OGA with the full p53 DNA binding domain.

### Different OGA Inhibitors Display Varying Levels of Peptide Mimicry

The three most potent and widely used OGA inhibitors are the transition state mimics PUGNAc (Haltiwanger et al., 1992) and GlcNAcstatin (Dorfmueller et al., 2010), as well as the NAG-thiazoline derivatives that mimic the oxazoline reaction intermediate (Macauley et al., 2005; Yuzwa et al., 2008). Thiazolines, such as Thiamet-G, only occupy the sugar pocket, whereas PUGNAc and GlcNAcstatin contain additional substituents mimicking the aglycon. Comparison with the *Cp*OGA-glycopeptide complexes reported here reveals that these phenyl moieties in fact occupy the +1/+2 subsites accommodating the glycopeptide backbone (Figure 2C). Since the use of PUGNAc and the thiazolines in probing the role of *O*-GlcNAc in modulating insulin sensitivity has yielded contradictory results (Macauley et al., 2008; Vosseller et al., 2002), further investigation is required to establish whether the different binding modes of these *O*-GlcNAcase inhibitors may explain the discrepancies between their effects in vitro.

### Conclusions

To our knowledge, this work reports the first structures of an enzyme of the *O*-GlcNAcase family in complex with glycopeptide substrates. Despite carrying different sequences, the glycopeptides adopt similar conformations in the active site. Crucially, while *O*-GlcNAcase does interact with the glycopeptide substrate backbone through specific enzyme side chains, the glycopeptide substrate side chains face away from the enzyme's binding cleft, explaining how a single enzyme can target a plethora of *O*-GlcNAc proteins. However, specific intramolecular interactions in the glycopeptide may predispose certain amino acid sequences for a conformation that is compatible with the *O*-GlcNAcase binding cleft. This work will underpin a mechanistic interpretation of differential cycling rates of sites in the *O*-GlcNAc proteome and facilitate development of inhibitors that not only target the sugar binding pocket, but also the peptide binding groove.

## Significance

**Protein O-GlcNAcylation is an essential and reversible glycosylation event in higher eukaryotes, where hundreds of intracellular proteins are O-GlcNAcylated. O-GlcNAc addition and removal is catalyzed by O-GlcNAc transferase and O-GlcNAcase, respectively. How a single pair of enzymes achieves cycling of the complete O-GlcNAc proteome is one of the key questions in the field. We report the molecular details of the interaction of a bacterial O-GlcNAcase with glycopeptide substrates, using three synthetic O-GlcNAc peptides matching established O-GlcNAc sites in the human proteome. In the 3D structures, we observe recognition of the sugar moiety as well as sequence-independent interactions with the peptide backbone, thus elucidating the molecular basis of the broad substrate specificity of the O-GlcNAcase enzyme. We report some influence of the peptide sequence directly surrounding the modification site; intramolecular hydrogen bonding within the peptide facilitates the binding to the enzyme. Peptides capable of forming such interactions are better O-GlcNAcase substrates in vitro, and we hypothesise that the cycling rate of individual O-GlcNAc sites in vivo may vary depending on the surrounding protein sequence. Finally, this work will aid development of O-GlcNAcase inhibitors that target the peptide binding site.**

## Experimental Procedures

### Glycopeptide Synthesis

Microwave-assisted solid phase peptide synthesis was performed with a CEM Liberty automated peptide synthesizer on low load Rink amide MBHA resin 100-200 mesh (Novabiochem) using standard Fmoc chemistry protocols on a 0.05 mmol scale. The 3,4,6**-**triacetyl-*O*-GlcNAc-Fmoc-Ser-OH building block was synthesized are described previously (Schimpl et al., 2010). All peptides were N-terminally acetylated and C-terminally amidated, and were purified via high-performance liquid chromatography.

### *Cp*OGA Expression

The previously reported pGEX6P1-*Cp*OGA_31−624_ construct (Rao et al., 2006) was truncated to *Cp*OGA_31−618_ by site-directed mutagenesis, and point mutations were introduced using the following oligonucleotide primers:

618stop 5′-caagaagctttaagttgagatttaacattaatatg 5′-catattaatgttaaatctcaacttaaagcttcttg-3′
D298N 5′-gcaatctattgggataatattcaagataagag 5′-ctcttatcttgaatattatcccaatagattgc-3′.

Recombinant *Cp*OGA was expressed as a glutathione S-transferase (GST) fusion in *E. coli* strain BL21(DE3)pLysS (Rao et al., 2006) and purified by glutathione sepharose affinity chromatography prior to proteolytic cleavage of the GST tag with PreScission protease. After desalting by dialysis, the protein was subjected to cation exchange chromatography on Q sepharose in 50 mM Bis-Tris (pH 6.4) with a linear 0-0.5 M NaCl gradient, and size exclusion chromatography on Superdex 75 resin in 25 mM Tris (pH 8.0), 150 mM NaCl.

### Crystallization and Structure Determination

*Cp*OGA D298N was concentrated to 35 mg/ml in 25 mM Tris/HCl (pH 8.0) and crystallized from 0.175 M CdSO_4_ and 0.6 M sodium acetate (pH 7.5) using sitting drop vapor diffusion. Glycopeptide complexes were achieved through soaking with 10 mM glycopeptide (see Schimpl et al., 2010) for glycopeptide synthesis) for 1-2 hr prior to cryoprotection with 20% glycerol in mother liquor. Diffraction data were collected at the European Synchrotron Radiation Facility (Grenoble, France) beam line ID14-4 and at Diamond Light Source (Didcot, UK) I03 (Table S1). Crystals belonged to space group P6_1_ and contained one molecule per asymmetric unit, with 72% solvent content. The structure was solved by molecular replacement, using the GlcNAcstatin C-complex of *Cp*OGA as a search model (Protein Data Bank ID 2J62), followed by iterative model building with COOT (Emsley and Cowtan, 2004) and refinement with REFMAC5 (Murshudov et al., 1997) using 2% of reflections as an R_free_ test set. Table S1 gives details of the data collection, processing, and refinement statistics.

### hOGA Expression and Purification

The coding sequence for full-length human OGA was cloned into pEBG6P. Mutations were introduced using the following oligonucleotide primers:

Y69F 5′-gtggtggaaggatttaaaggaagaccttggg 5′-cccaaggtcttcctttaaatccttccaccac-3′
Y69K 5′-gtggtggaaggaaagaaaggaagaccttggg 5′-cccaaggtcttcctttctttccttccaccac-3′
Y69Q 5′-gtggtggaaggacagaaaggaagaccttggg 5′-cccaaggtcttcctttctgtccttccaccac-3′
Y69S 5′-gtggtggaaggatcgaaaggaagaccttggg 5′-cccaaggtcttcctttcgatccttccaccac-3′.

Protein was expressed in transiently transfected HEK293 cells and purified via glutathione sepharose affinity chromatography.

### Enzymology

hOGA and *Cp*OGA glycopeptide hydrolysis assays were carried out as described previously (Dorfmueller et al., 2006; Schimpl et al., 2010) using multisubstrate enzyme kinetics with the fluorigenic pseudosubstrate 4MU-GlcNAc as the reporter substrate. Briefly, initial rates of hydrolysis of 4MU-GlcNAc were determined in the presence of increasing concentrations of glycopeptide, and the Michaelis constant of the competing substrate (*K*_m_′) was determined using the following equation:

$$\frac{\upsilon_{i}}{\upsilon_{0}}=\frac{1+\frac{K_{m}}{S}}{1+\frac{K_{m}}{S}\left(1+\frac{S^{\prime }}{K_{m}^{\prime }}\right)},$$

wherein *v*_i_/*v*_0_ is the relative activity in the presence of inhibitor, *K*_M_ and *S* are the Michaelis constant and substrate concentration of the reporter substrate, and *S*′ is the concentration of glycopeptide. Reactions were performed at 37°C in 50 mM citrate-phosphate buffer (pH 7.4) and 0.1 mg/ml BSA. Experiments were performed in triplicate, and data were analyzed and plotted with GraphPad PRISM.

## Figures and Tables

**Table 1:** Hydrophobic Stacking Interactions of Tyr69 Are Essential for Substrate Binding by hOGA

	TAB1 Peptide	hOGA Peptide	p53 Peptide	p53 Peptide
	PVSVPYS(*O-*GlcNAc)SAQSTS	VAHS(*O-*GlcNAc)GAK	VDS(*O-*GlcNAc)TPG	QLWVDS(*O-*GlcNAc)TPPPG
Y69S	>4,000	>4,000	3,400 ± 500	290 ± 20
Y69K	>4,000	>4,000	>4,000	350 ± 20
Y69Q	3,600 ± 300	>4,000	>4,000	170 ± 40
Y69F	1,100 ± 60	1,200 ± 110	2,700 ± 400	53 ± 5
Wild-type	940 ± 80	790 ± 40^a^	1,300	21 ± 3

Tyr69 in human OGA may participate in hydrogen bonding with the catalytic aspartate (Asp175) as well as providing a stacking platform for the −1/−2 peptide bonds in the substrate. The Y69S mutation disrupts both these interactions, the Y69K and Y69Q mutants cannot participate in pi-pi stacking, and Y69F abolishes the hydrogen bond. Point mutations were introduced in hOGA, and the Michaelis constant (*K*_M_, given in μM) was determined for several peptide substrates.

a Schimpl et al., 2010.

**Table 2:** Intramolecular Hydrogen Bonds Stabilize the Substrate Conformation and Reduce the *K*_m_ for hOGA

	TAB1 Peptide (Ser395)	p53 Peptide (Ser149)
Original sequence	VPYS(*O-*GlcNAc)SAQ	QLVDS(*O-*GlcNAc)TPPPG
* K*_M_ (hOGA)	4,100 ± 300 μM	21 ± 2 μM
Altered sequences	VP**H**S(*O-*GlcNAc)SAQ	QLVVS(*O-*GlcNAc)**V**PPPG
* K*_M_ (hOGA)	810 ± 140 μM	3,700 ± 900 μM
		QLV**V**S(*O-*GlcNAc)TPPPG
* K*_M_ (hOGA)		1,800 ± 200 μM

The contribution of intramolecular hydrogen bonds toward substrate binding was probed by introducing a potential hydrogen bond donor in the sequence of the TAB1 peptide. For the p53 peptide, the hydrogen bond was disrupted by replacing either the donor or the acceptor with isosteric aliphatic amino acids (highlighted in boldface and underscored).
