# Supplementary material for: Synergy of Peptide and Sugar in O-GlcNAcase Substrate Recognition
Source: Chem Biol. 2012 Feb 24;19(2):173–8. doi: 10.1016/j.chembiol.2012.01.011 (PMC3476531; doi:10.1016/j.chembiol.2012.01.011)
Supplement: Document S1. Figures S1 and S2 and Table S1 [file mmc1.pdf]

Chemistry & Biology, Volume *19*

Supplemental Information

Synergy of Peptide and Sugar in *O*-GlcNAcase Substrate Recognition

Marianne Schimpl, Vladimir S. Borodkin, Lindsey J. Gray, and Daan M.F. van Aalten

<u>Inventory of Supplemental Information</u>

Fig. S1 Human and bacterial OGA show similar K_m trends for a range of glycopeptide substrates.
Related to Fig. 1B, depicting the correlation between Km values for different substrate peptides for the human enzyme and the bacterial homologue.

Fig. S2 Accessibility of the active site in the new crystal form of *Cp*OGA.
Related to Fig. 1C-D, demonstrating the advantages of the crystal form reported here, and its applicability for soaking experiments.

Table S1. Data collection and refinement statistics for *Cp*OGA D298N in complex with GlcNAc-modified peptides.
Related to and referenced in the Experimental Procedures section.

Figure S1. Human and bacterial OGA show similar K_m trends for a range of glycopeptide substrates. (Related to Figure 1B). Correlation plot of the K_m values of glycopeptide substrates for hOGA and *Cp*OGA. Numeric values of the data plotted in here as well as in Fig. 1B are given below.

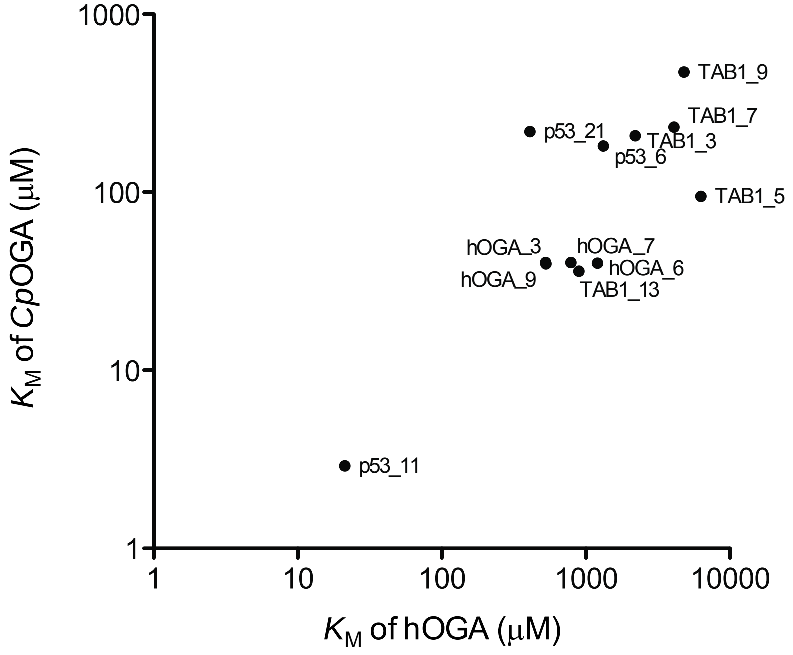

K_m (μM)<br>Peptide length (a.a.)	hOGA	*Cp*OGA
TAB1 (Ser395)		
3	2200 ± 100	210 ± 30
5	6300 ± 500	100 ± 20
7	4100 ± 300	230 ± 20
9	4800 ± 300	470 ± 70
13	900 ± 30	36 ± 1
hOGA (Ser405)		
3	520 ± 10	40 ± 5
6	1200 ± 40	40 ± 10
7	790 ± 40	40 ± 10
9	530 ± 20	40 ± 6
p53 (Ser149)		
5	1300 ± 200	48 ± 8
11	21 ± 2	2.9 ± 0.4
21	410 ± 20	220 ± 10

Figure S2. Accessibility of the active site in the new crystal form of *Cp*OGA. (Related to Fig. 1C-D)

In the previously published $I2_12_12_1$ crystal form (PDB ID 2J62) of *Cp*OGA, crystal contacts limit the accessibility of the enzyme's active site. The new $P6_1$ crystal form reported here possesses a similar protein conformation (RMSD of 0.50 Å over 578 C_αs) but fully accessible active site. In the figure below, one molecule of *Cp*OGA is depicted with α-helices shown in cyan and β-sheets shown in blue, with a semi-transparent surface. Neighboring symmetry-related protein molecules are shown in shades of grey. The 11-amino acid glycopeptide (pink sticks) is bound in the active site, which is facing a solvent channel, making this crystal form well suited for soaking experiments. The shortest distance between the glycopeptide and any atom on a symmetry-related protein molecule is 9 Å.

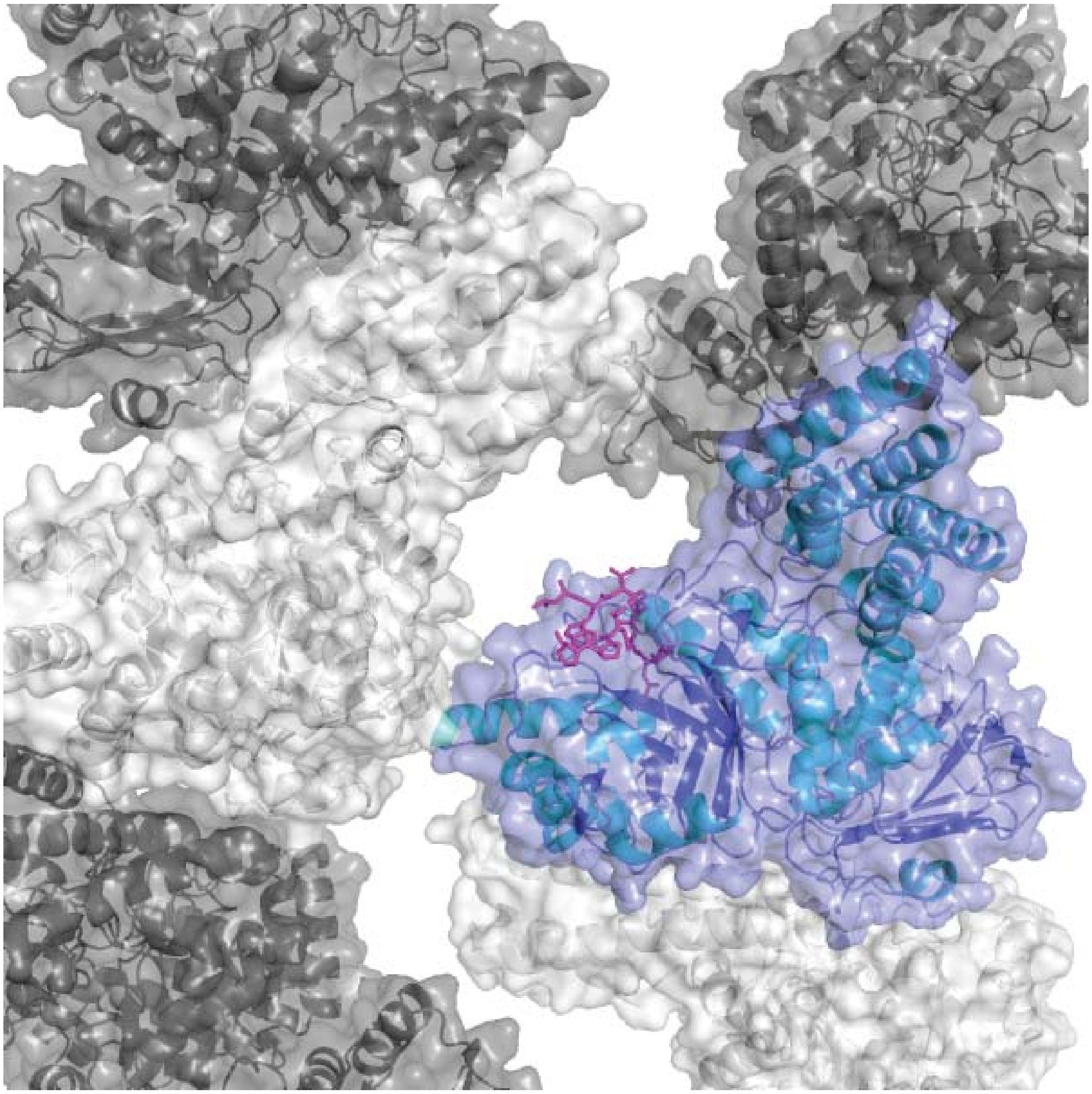

Table S1. Data collection and refinement statistics for *Cp*OGA D298N in complex with GlcNAc-modified peptides. Values for the highest resolution shell are given in parentheses.

	*Cp*OGA D298N : hOGA O-GlcNAc peptide Ac-VAHS(*O*-GlcNAc)GA-NH_2	*Cp*OGA D298N : TAB1 O-GlcNAc peptide Ac-VPYS(*O*-GlcNAc)SAQ-NH_2	*Cp*OGA D298N : p53 O-GlcNAc peptide Ac-QLWVDS(*O*-GlcNAc)TPPPG-NH_2
**Data collection**			
Beamline, wavelength	ID14-1, 0.934 Å	I03, 0.976 Å	I03, 0.976 Å
Space group	$P6_1$	$P6_1$	$P6_1$
Cell dimensions (Å)	*a*=*b*=118.1, *c*=148.2	*a*=*b*=118.1, *c*=148.3	*a*=*b*=118.1, *c*=148.3
Resolution (Å)	25-2.6 (2.69-2.60)	24.6-2.55 (2.69-2.55)	25-2.75 (2.90-2.75)
R_{merge}	0.155 (0.679)	0.122 (0.667)	0.154 (0.575)
$I/\sigma I$	6.9 (2.5)	7.1 (2.0)	7.5 (2.7)
Completeness (%)	100.0 (100.0)	98.7 (99.4)	92.5 (94.3)
Redundancy	7.7 (6.8)	3.1 (3.1)	4.4 (4.3)
**Refinement**			
Unique reflections	36085	37698	28111
R_{work}, R_{free}	0.197 / 0.235	0.190 / 0.226	0.185 / 0.219
No. atoms			
Protein	4578	4580	4578
Cadmium	19	19	19
Glycopeptide	55	57	77
Water	77	122	102
B-factors			
Protein	27	36	28
Glycopeptide	40	59	48
R.m.s. deviations			
Bond lengths (Å)	0.013	0.013	0.014
Bond angles (°)	1.45	1.44	1.46
Ramachandran plot			
most favoured (%)	90.2	90.3	90.0
generously allowed (%)	9.1	9.3	9.2
additionally allowed (%)	0.8	0.4	1.0
disallowed (%)	0.0	0.0	0.0
**Pdb ID**	**2ydq**	**2yds**	**2ydr**
